# Recurrent polychondritis complicated with demyelinating encephalopathy and limbic encephalitis: a case report and literature review

**DOI:** 10.3389/fmed.2026.1825793

**Published:** 2026-05-04

**Authors:** Xinyue Wang, Yue Wang, Ping Lin

**Affiliations:** 1 Department of Neurology, Xinhua Hospital Affiliated to Dalian University, Dalian, Liaoning, China; 2 Department of Medical Psychology, No. 967 Hospital, Joint Logistics Support Force, Chinese People's Liberation Army (Maolin Campus), Dalian, Liaoning, China

**Keywords:** autonomic dysfunction, cartilage inflammation, demyelination, neuroinflammation, rheumatic disease

## Abstract

Recurrent polychondritis (RP) is a rare systemic autoimmune disorder that rarely involves the central nervous system (CNS). This report describes a 68-year-old male RP patient who was admitted with bilateral auricular swelling and pain that had lasted for one month, accompanied by limb weakness and pain that had lasted for ten days. Neurological assessment revealed sluggish responses, dysarthria, decreased limb muscle strength and urinary/bowel dysfunction, with a Montreal Cognitive Assessment (MoCA) score of 21/30. Laboratory tests showed elevated inflammatory markers, and autoantibodies in the serum and cerebrospinal fluid (CSF) were negative. CSF analysis revealed increased intracranial pressure and elevated protein levels, as well as decreased immunoglobulin and complement levels. A brain MRI revealed bilateral periventricular, centrum semiovale, and medial temporal lobe fluid-attenuated inversion recovery (FLAIR) hyperintensities. The diagnosis of RP was confirmed based on the Damiani–Levine criteria, alongside the diagnoses of autoimmune limbic encephalitis (LE) and secondary demyelinating encephalopathy (DE). Following treatment with methylprednisolone pulse therapy followed by cyclophosphamide, the patient’s symptoms improved significantly. At the three-month follow-up, limb muscle strength had recovered, but urinary dysfunction persisted. This case demonstrates that RP can affect the CNS and the central-autonomic axis. Early recognition and initiation of immunotherapy can improve prognosis.

## Introduction

Relapsing polychondritis (RP) is a rare systemic autoimmune disease that primarily affects cartilage-rich structures, such as the ears, nose, throat, trachea and joints (1). Central nervous system (CNS) involvement is uncommon in RP, occurring in around 3% of cases. Previous reports have primarily described aseptic meningitis, limbic encephalitis (LE), autoimmune encephalitis, ischaemic stroke, myelitis, and cranial nerve disorders (2–8). LE is a subacute autoimmune inflammatory syndrome predominantly affecting the medial temporal lobes, characterized by memory impairment, psychiatric symptoms, and seizures, and can be diagnosed even in the absence of identifiable neuronal antibodies according to established clinical criteria (9). Demyelinating encephalopathy (DE) is a descriptive term denoting inflammatory demyelination of the central nervous system (10). This entity should be distinguished from acute disseminated encephalomyelitis (ADEM), which is typically a monophasic immune-mediated disorder associated with a preceding infection or vaccination, whereas the present case reflects a secondary demyelinating process of the central nervous system in the context of RP. Cases of RP complicated by DE, LE and damage to the central autonomic nervous system have never been reported before. This report describes a patient with RP presenting with multisystem neurological dysfunction, including rapidly progressive limb weakness, cognitive impairment, and urinary and fecal incontinence.

## Case presentation

A 68-year-old male patient was admitted to our hospital, due to “bilateral auricular swelling and pain for 1 month, and weakness with pain in limbs for 10 days.” He had a 10-year history of hypertension and a history of brainstem infarction, with residual mild weakness of the right upper and lower limbs. He took aspirin and statins for a long time and denied a family history of genetic diseases. The patient received antibiotics combined with low-dose glucocorticoids for auricular swelling and pain in another hospital, but the symptoms were not relieved. Subsequently, he developed limb weakness that progressed to inability to walk, accompanied by dysarthria and bradyphrenia. The other hospital found no evidence of infarction; after symptomatic treatment, the limb weakness did not improve, so he was transferred to our hospital for further treatment. Urinary and fecal retention were present during the disease course. The patient denied any prior episodes of auricular chondritis.

### Physical examination

**Figure 1:**
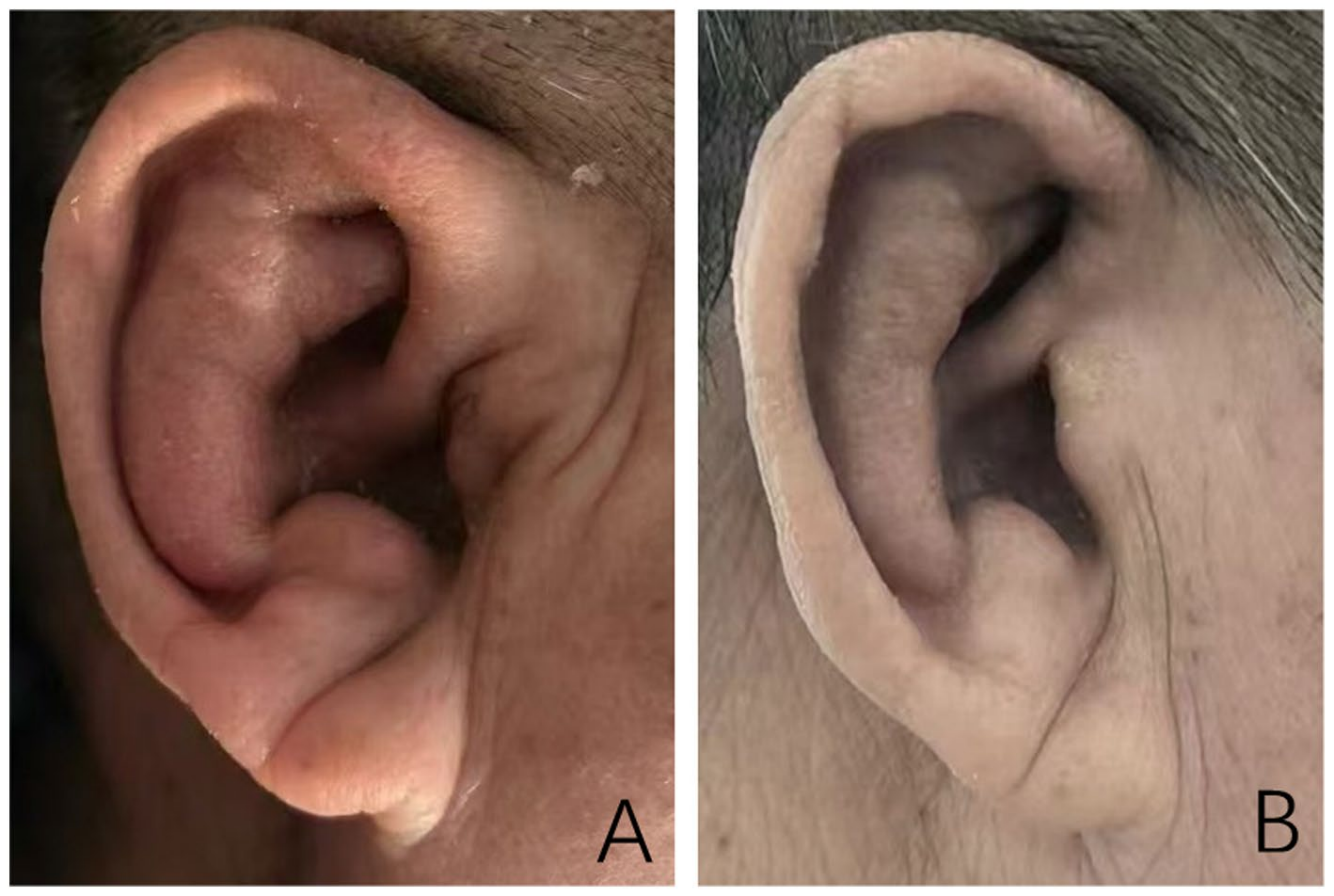
**(A)** At the time of onset, the auricle was markedly swollen, and the earlobe was not involved. **(B)** After hormone treatment, the swelling of the auricle disappeared.

Temperature 37.1 °C, blood pressure 143/89 mmHg, bilateral auricular swelling (earlobes not involved) (Figure 1), and bilateral hand arthralgia without swelling or deformity. No nasal redness, swelling, or tenderness was observed, and there was no conjunctival injection or visual impairment; the patient reported no tinnitus, hearing loss, or vertigo.

### Neurological examination

Clarity of mind and bradyphrenia, dysarthria, bilateral miosis (D = 1 mm), hyperalgesia of the face and limbs; increased muscle tone of the right upper limb, with muscle strength of grade 3 in the right upper limb, grade 5- in the left upper limb, grade 1 in the right lower limb, and grade 2 in the left lower limb; hyperreflexia of bilateral tendons, positive bilateral palmomental reflex and Babinski sign; MoCA score was 21/30, with urinary and fecal retention.

### Auxiliary examinations

**Figure 2:**
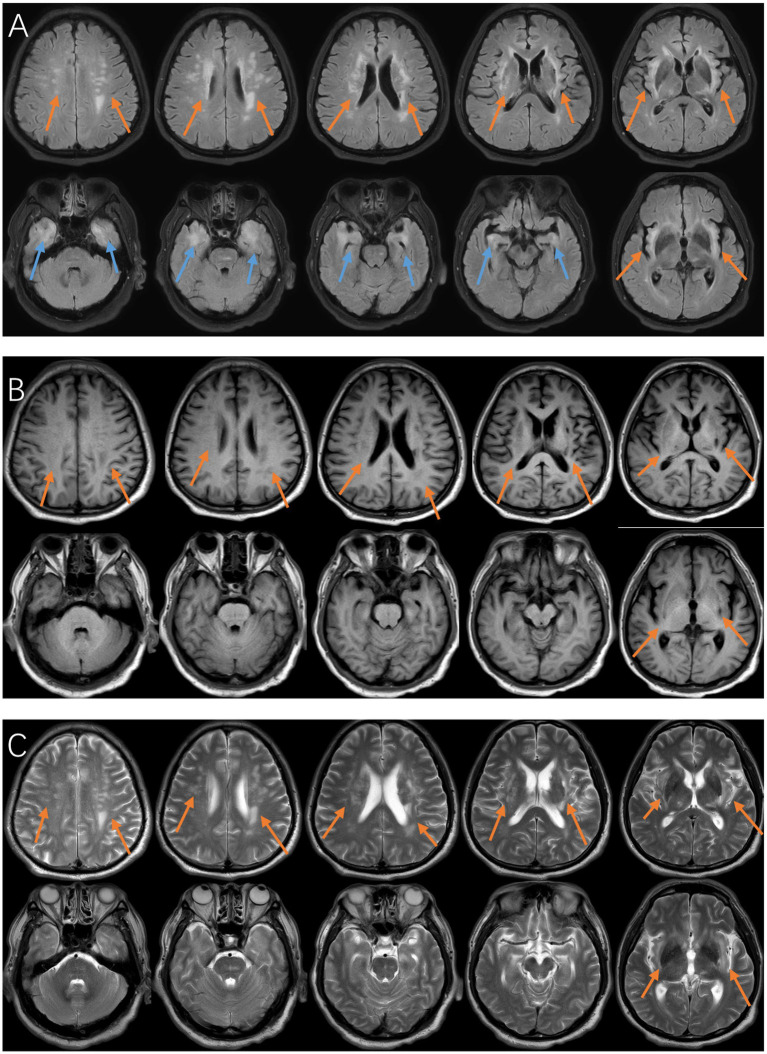
**(A)** FLAIR, orange arrows show patchy periventricular and centrum semiovale hyperintensities (demyelinating changes); blue arrows show bilateral medial temporal lobe hyperintensities (limbic encephalitis changes). **(B)** T1, arrows show patchy hypointensities in the periventricular and centrum semiovale regions, indicating underlying tissue injury associated with demyelination. **(C)** T2, arrows show patchy hyperintensities in the periventricular and centrum semiovale regions, further delineating lesion extent and reflecting increased water content. The combined use of FLAIR, T1, and T2 sequences provides complementary information regarding lesion distribution, extent, and severity.

Inflammatory indicators [C-reactive protein (CRP) 86.4 mg/L, (Erythrocyte sedimentation rate) ESR 65 mm/h] and coagulation indicators were elevated; blood routine showed increased neutrophil ratio and decreased lymphocyte count, with mild abnormal liver function; all relevant antibodies were negative (Table 1). CSF examination showed increased intracranial pressure (330 mmH₂O), elevated nucleated cell count and protein level, and decreased immunoglobulin and complement levels (Table 2). A brain MRI performed 1 day after admission revealed bilateral periventricular and centrum semiovale T2-weighted fluid-attenuated inversion recovery (T2-FLAIR) hyperintensities and T1 hypointensities, as well as bilateral medial temporal lobe FLAIR hyperintensities (Figure 2); chest Computed tomography (CT), limb nerve conduction velocity, and arteriovenous examinations were unremarkable. Due to poor cooperation, a complete spinal MRI could not be performed; MRI of the cervical and lumbar spine showed no abnormal spinal cord signal.

**Table 1:** Summary of laboratory test results.

Laboratory parameters	Result	Unit	Reference range
Inflammation and coagulation markers			
C-reactive protein (CRP)	86.4↑	mg/L	0–8
D-dimer	5.63↑	ug/mL	0–0.5
Fibrinogen	6.84↑	g/L	2–4
Fibrinogen degradation products (FDP)	30.66↑	ug/mL	0–5
Erythrocyte sedimentation rate (ESR)	65↑	mm/h	0–15
Liver function and nutritional status			
Gamma-glutamyl transferase (GGT)	56.3↑	U/L	11–50
Aspartate aminotransferase (AST)	43.3↑	U/L	0–40
Alanine aminotransferase (ALT)	58.6↑	U/L	9–50
Albumin (ALB)	37.9↓	g/L	40–55
Complete blood count			
Neutrophil percentage	79.1↑	%	40–75
Lymphocyte percentage	11.5↓	%	20–50
Absolute lymphocyte count	0.88↓	×10^9^/L	1.1–3.2
Thyroid function			
FT₄	25.97↑	pmol/L	11.2–23.81
TSH, TT₄, TT₃, FT₃, TPOAb	Within normal range		
Immunological indicators			
Complement C3	1.59↑	g/L	0.7–1.4
Complement C4, IgA, IgG, IgM	Within normal range		
Autoantibody panel			
ANA, dsDNA, SSA, SSB, Sm, RNP, Jo-1, Scl-70, Ro-52, ACA, AMA, AHA, ANuA, PM-Scl, RF, Anti-O, Anti-CCP	Negative		
Tumor Markers (NSE, CEA, AFP)	Within normal range		
Central nervous system demyelinating disease antibodies (4 items)			
AQP4, MOG, MBP, GFAP antibodies (IgG)	Negative		
Autoimmune encephalitis antibody panel (16 items)			
MOG, GFAP, GABAARα1, GABAARβ3, NMDAR GluN2A, NMDAR GluN2B, mGluR1, mGluR2, Neurexin-3α, Cavα2δ, KLHL11, GluK2, PDE10A, AK5, KCTD16, NCAM1 antibodies (IgG)	Negative		

**Table 2:** Summary of CSF examination results.

Parameter	Result	Reference range
Opening pressure	330 mmH₂O↑	80–180
Appearance	Pale yellow, clear	Colorless and transparent
Nucleated cell count	24 × 10^6^/L↑	0–8
Lymphocyte percentage	81.5%↑	60–80%
Glucose	3.2 mmol/L	2.2–3.9
Chloride	117 mmol/L↑	99–110
CSF protein	0.76 g/L↑	0.12–0.60
IgA	<0.2 g/L↓	1.0–4.1
IgG	0.31 g/L↓	8.6–17.4
IgM	<0.05 g/L↓	0.3–2.2
Complement C3	<0.04 g/L↓	0.7–1.4
Complement C4	<0.02 g/L↓	0.1–0.4
Bacterial smear, acid-fast bacilli smear, and CSF culture	No abnormalities detected; no bacterial growth	
Central nervous system demyelinating disease antibodies (4 items)		
AQP4; MOG; MBP; GFAP antibodies (IgG)	Negative	
Autoimmune encephalitis antibody panel (16 items)		
MOG; GFAP; GABAARα1; GABAARβ3; NMDAR GluN2A; NMDAR GluN2B; mGluR1; mGluR2; Neurexin-3α; Cavα2δ; KLHL11; GluK2; PDE10A; AK5; KCTD16; NCAM1 antibodies (IgG)	Negative	

### Diagnosis

According to the Damiani–Levine diagnostic criteria (11), the patient presented with bilateral auricular chondritis and non-erosive arthritis of both hands, both showing a good response to glucocorticoid therapy, confirming the diagnosis of RP. The patient exhibited bradyphrenia with impaired memory and calculation, and a MoCA score of 21/30. CSF analysis showed a nucleated cell count >5 × 10^6^/L (predominantly lymphocytes), and FLAIR demonstrated bilateral hyperintense lesions confined to the medial temporal lobes, confirming the diagnosis of autoimmune-LE (9). The patient also had neurological deficits, with bilateral palmomental reflexes (+) and Babinski signs (+), while electromyography (EMG) and electroneurography (ENG) excluded peripheral neuropathy. T2-FLAIR showed patchy hyperintense lesions in the bilateral periventricular regions and centrum semiovale, with corresponding hypointensity on T1-weighted images. Lumbar puncture showed elevated CSF protein levels with markedly reduced immunoglobulins and complement, suggesting immune consumption. These findings support a diagnosis of RP-associated inflammatory demyelinating encephalopathy. Cerebrospinal fluid immunoglobulin levels were decreased rather than elevated, whereas intrathecal IgG synthesis occurs in approximately 90–95% of patients with Multiple Sclerosis (12). Although oligoclonal bands were not assessed, the overall CSF profile does not support intrathecal IgG synthesis, making MS less likely. The favorable response to glucocorticoids further supports an immune-mediated mechanism and suggests potential reversibility.

### Treatment and outcome

The patient was given methylprednisolone pulse therapy with gradual tapering, which was then switched to oral prednisone combined with cyclophosphamide. At discharge, the patient showed improved limb weakness and could stand with assistance. Limb pain had resolved, and bilateral auricular swelling and tenderness had subsided. MoCA score of 25/30. Muscle strength was 5/5 in the upper limbs and 4/5 in the lower limbs, while urinary and fecal dysfunction persisted. After discharge, he continued regular medication, gradual tapering of glucocorticoids and rehabilitation treatment. At 3 months of follow-up, the patient’s limb muscle strength returned to normal, defecation function recovered, but urinary retention persisted; bilateral pupils recovered to 1.5 mm, and bilateral Babinski’s sign and palmomental reflex remained positive throughout the disease course and were still present at follow-up (Table 3).

**Table 3:** Clinical symptoms, laboratory findings, and treatment details before and after immunosuppressive therapy.

Category		Timeline				Normal range	Units
		10 days pre-IT	0 days IT	9 days post-IT	3 months post-IT		
Clinical symptom	Auricular swelling	Y	Y	N	N	N/A	N/A
	Bilateral pupils	-	D = 1	D = 1	D = 1.5	2–5	mm
	Dysarthria	Y	Y	N	N	N/A	N/A
	Limb weakness	Y (Not evaluated)	R-U 3/5, L-U 5^−^/5, R-L 1/5, L-L 2/5.	R-U 5/5, L-U 5/5, R-L 4/5, R-L 4/5.	R-U 5/5, L-U 5/5, R-L 5/5, R-L 5/5.	5	Grade
	Limb pain	Y	Y	N	N	N/A	N/A
	Urination disorder	Y	Y	Y	Y	N/A	N/A
	Defecation disorder	N	Y	Y	N	N/A	N/A
	Babinski sign(+)	Y	Y	Y	Y	N/A	N/A
	Cognitive impairment (MoCA score)	-	21	25	26	26–30	Score
laboratory findings	WBC	-	7.68	11.10	-	3.5–9.5	×10^9^/L
	CRP	-	86.4	<6	-	0–10	mg/L
	ESR	-	65	20	-	0–15	mm/h
	D-dimer	-	5.63	3.36	-	0–0.5	ug/mL
	AST	-	43.3	23.2	-	0–40	U/L
	ALT	-	58.6	60.4	-	9–50	U/L
IT	Methylprednisolone	-	500 → 250 → 125 mg/day IV, each × 3 days		60 mg/day oral, taper 5 mg/week until discontinuation		
	Cyclophosphamide	N	N	N	100 mg oral, every alternate day		

IT, immunosuppressive therapy; WBC, white blood cells; CRP, C-reactive protein; ESR, erythrocyte sedimentation rate; AST, Aspartate aminotransferase; ALT, Alanine aminotransferase; Y, yes; N, no; N/A, not applicable; D, diameter; R-U, Right-Upper limb; L-U, Left-Upper limb; R-L, Right-Lower limb; L-L, Left-Lower limb; IV, intravenous; −, no record available.

## Discussion

RP is a rare systemic autoimmune disease with an unclear pathogenesis and a lack of specific diagnostic markers (1). Current evidence suggests that its development is mainly related to autoimmune abnormalities and molecular genetic factors, including autoantibodies against type II collagen (13), matrilin-1 (14), and type IX and XI collagen (15), as well as somatic mutations in the UBA1 gene (16). CNS involvement in RP is uncommon. Previous studies (17) suggest that the most likely mechanism is secondary CNS vasculitis associated with systemic vasculitis, leading to neurological complications resembling cerebral vasculitis. Recent reports have also indicated that in patients with RP complicated by anti-gamma-aminobutyric acid B receptor (GABA_B receptor) antibody–associated LE, antigen cross-reactivity and disturbances in GABA metabolism may contribute to CNS injury, providing theoretical support for CNS involvement in RP (2). However, no specific immune-related antibodies have yet been identified for CNS involvement in RP, and the underlying mechanisms remain to be clarified.

Husein et al. (4) previously reported a case of seronegative LE associated with RP, in which multiple common autoimmune encephalitis–related antibodies (including NMDAR, Glycine, LGI1, CASPR2, IgLON5, AMPA1/2, GABA_A, and DPPX antibodies) were not detected. In the present case, 16 autoimmune encephalitis–related antibodies in serum and CSF, as well as four antibodies associated with central demyelinating diseases, were also negative. A Chinese study including 181 patients with RP showed that those with CNS involvement tended to be older at admission (8). Encephalitis was the most common manifestation, followed by aseptic meningitis and meningoencephalitis, and some patients exhibited multiple neurological involvements. Most patients demonstrated elevated intracranial pressure and CSF protein levels with lymphocyte-predominant inflammatory responses, and nearly all patients with CNS involvement had concomitant auricular involvement (8). Zhu et al. (18) reported a case of RP with persistent white-matter injury detected 15 months after treatment. In the present case, CNS involvement manifested as DE combined with LE and damage to the central-autonomic axis. Compared with previous reports, this case simultaneously presented two rare CNS manifestations with complex clinical features.

The association between DE and RP remains poorly characterized; however, accumulating evidence from other systemic autoimmune diseases suggests that immune-mediated mechanisms may underlie central nervous system demyelination (10, 19). Studies in conditions such as systemic lupus erythematosus and other rheumatic diseases have shown that autoimmune dysregulation can target myelin and lead to inflammatory demyelination (20, 21). Mechanistically, molecular mimicry, blood–brain barrier disruption, and intrathecal immune activation may contribute to myelin injury (22). In the present case, the acute to subacute course, systemic autoimmune background, and imaging features suggestive of inflammatory demyelination point toward an immune-mediated process rather than a primary degenerative, metabolic, or vascular white matter disorder. The observed clinical improvement following corticosteroid therapy further supports this interpretation. Therefore, the white matter lesions are more likely part of RP-related central nervous system involvement rather than an independent leukoencephalopathy.

Autonomic nervous system involvement is not a typical manifestation of RP, and reports of RP associated with autonomic dysfunction are currently lacking. The central autonomic network (CAN) comprises several key structures, including the cerebral cortex (such as the insular cortex and anterior cingulate gyrus), the limbic system (amygdala and hippocampus), the hypothalamus, brainstem nuclei (e.g., the dorsal motor nucleus of the vagus and locus coeruleus), and the spinal autonomic centers, which together regulate visceral activity and autonomic function (23). In addition to affecting cognition and psychiatric function, LE may involve the CAN and lead to central autonomic dysfunction. In this case, the patient presented with bilateral miosis, urinary and fecal dysfunction, and widespread hyperalgesia. Bilateral miosis may suggest sympathetic dysfunction, consistent with impaired autonomic regulation underlying the urinary and fecal dysfunction. EMG and ENG showed no evidence of peripheral neuropathy, supporting a central autonomic network involvement. Decreased immunoglobulin and complement levels in the CSF may reflect immune consumption, suggesting ongoing immune activation and intrathecal immune dysregulation. Although nonspecific, this finding may support an active immunopathological process associated with central nervous system involvement in RP. Clinically, when patients with RP develop unexplained autonomic dysfunction, involvement of the central-autonomic axis should be considered. Because autonomic dysfunction often recovers slowly and may be severe, it may represent a severe phenotype of RP-related CNS involvement.

A study published in Nature in 2024 reported that in certain RP subtypes, such as VEXAS syndrome–associated RP and MAGIC syndrome, CNS involvement is more common and frequently accompanied by thromboembolism, vasculitis, and marked systemic inflammation (24). These findings suggest that CNS involvement in RP may represent a severe disease phenotype or reflect a specific molecular immunological background with important clinical implications. RP with CNS involvement is often characterized by multisystem damage and high inflammatory burden and is closely associated with severe involvement of the heart, eyes, and ears, representing a high-risk subtype with poor prognosis (8). In the present case, treatment with methylprednisolone pulse therapy combined with cyclophosphamide resulted in improvement in inflammatory markers and partial recovery of neurological function, indicating that early immunotherapy may improve prognosis. Clinically, when patients with RP develop unexplained cognitive impairment, limb weakness, or autonomic dysfunction, CNS involvement should be strongly suspected, and prompt immunotherapy and rehabilitation interventions should be initiated to improve both short- and long-term outcomes.

## Conclusion

This case report describes a patient with RP complicated by CNS damage, accompanied by LE, DE, and clinical symptoms of autonomic nerve damage, suggesting that RP can involve the CNS and the central-autonomic nerve axis. Clinically, for patients with unexplained neurological dysfunction accompanied by auricular inflammation, high vigilance should be paid to CNS involvement by RP; early immunotherapy and comprehensive rehabilitation can improve the prognosis.

## Data Availability

The original contributions presented in the study are included in the article/supplementary material, further inquiries can be directed to the corresponding author.
